# Newborn Screening for Lysosomal Disease: Mission Creep and a Taste of Things to Come?

**DOI:** 10.3390/ijns4030021

**Published:** 2018-06-27

**Authors:** Bridget Wilcken

**Affiliations:** 1 Centre for Clinical Genetics, Sydney Children’s Hospital, Randwick, NSW 2031, Australia; bridget.wilcken@health.nsw.gov.au; Tel.: +61-293-825-609; 2 Paediatrics and Child Health, University of Sydney, Sydney, NSW 2006, Australia

**Keywords:** newborn screening, outcome, lysosomal storage disorders, Pompe disease, Krabbe disease, mission creep

## Abstract

Newborn screening for several lysosomal disorders can now be accomplished successfully for case finding. However, many cases identified do not require immediate intervention and it is not yet clear, for some disorders, if there is a benefit in early diagnosis for those cases, or what should be called a benefit. Diagnosing adult-onset cases, especially when there are quite imperfect genotype-phenotype correlations, represents a significant expansion of what has heretofore been considered the aim of newborn screening. This mission creep should be carefully discussed, and certain aspects of newborn screening strengthened. We should all proceed with caution in this field.

## 1. Introduction

The major factors driving the addition of disorders to newborn screening programmes are technological advances in testing and, more importantly, the development of effective treatments that require early implementation. In recent years, the pace of development of new methodologies and new treatments has increased dramatically, and this will greatly increase the number of new candidate disorders for screening. This is a harbinger of mission creep—significantly expanding and changing the currently expressed aims of newborn screening.

The promise of treatment for LSDs (lysosomal storage disorders) was evident over 50 years ago and effective treatment for a few disorders was present in the late 1990s, as were tests suited to mass screening, so it is unsurprising that there has been strong interest in newborn screening. The first actual mention of enzyme replacement therapy was in Science in 1970 [1], when two Fabry hemizygotes were infused with normal plasma. Enzyme activity increased rapidly (but faded quite fast). The authors concluded that their experiment supported the hypothesis that, “enzyme replacement therapy by plasma infusion will be a means of therapy for this glycosphingolipidosis.” In the last two decades, enzyme replacement therapy (ERT)—delivering the appropriate enzyme by intravenous infusion at intervals, commonly fortnightly—has indeed become the major treatment for several LSDs, although some tissues such as brain, cartilage, and bone are quite refractory to treatment. There is an enormous associated cost.

Many early papers also discussed possible screening, initially by using urine-based tests. The first publications to discuss methods suited to dried blood spot screening came in the 1990s when John Hopwood’s group in Adelaide looked at possible marker proteins, and in 1999 reported a trial of screening some 11,000 neonates for the lysosomal-associated membrane protein LAMP1 [2] and later suggested a two-tier approach. This methodology, although seemingly successful, was quickly overtaken when Chamoles published in 2001 a method for measuring the enzyme, alpha-l-iduronidase, in dried blood spots (DBS), for screening mucopolycaccharidosis type I [3]. He followed quickly in that year and the next with enzymatic methods from DBS for seven other disorders, using bench-top fluorimetric methods. In 2004, Michael Gelb published a tandem mass-spectrometry method for multiplex testing for five disorders [4]; a seminal paper that prompted widespread consideration of testing. Another method now in use is digital microfluidics fluorometry [5], and there is a robust debate about the relative advantages of these two [6]. In any case it has been apparent for some time that we *can* screen for LSD, with technology that measures biochemical and molecular markers, and that there will be an expansion of the numbers of diseases that can be treated at some level. The more interesting and important question is whether, for some of these disorders, we should screen.

The criteria for screening are usually based on the excellent WHO guidance from Wilson and Jungner [7], although this had little specifically about newborn screening (only phenylketonuria screening was taking place) and the criteria were subjective. Later modifications and published criteria have not quite kept up with current screening development, let alone the likelihood of next-generation sequencing becoming, in some form, a primary test (e.g., [8]). Attitudes may now be changing about what is desirable to include in a newborn screening programme, which certainly poses new ethical dilemmas. There is broad agreement that benefits from screening should outweigh harms, but little or no discussion as to what actually is a benefit (and who could be the beneficiary), what are the possible harms, and how these may be measured.

In the early days of screening, perceived harms were linked mainly to screening test performance—the effects of false positive and false negative test results, costs (opportunity costs), and in some jurisdictions, the availability of appropriate treatment. Perceived benefits were medical benefits to the baby screened—reduced mortality and morbidity in those found to be affected. But things have proved much more complex. Almost all forms of screening detect more cases than expected, some “extra” cases being very mild and clinically insignificant [9]. Looming large are two issues that have come to light principally with LSD newborn screening: Firstly, for several LSD disorders, distinguishing among detected “cases” which ones are early onset, which are late onset, which are clinically insignificant biochemical variations, and which are simply carriers has proved difficult. Thus, screening is diagnosing babies as apparently affected who may develop the disorder much later, perhaps in adulthood, or perhaps never. These are the “patients in waiting” of Timmermans and Buchbinder [10]. In LSD a preponderance of late-onset phenotypes is proving the norm. The second looming issue is the absence of robust discussion about outcomes: what constitutes an overall advantage, what is an acceptable outcome? Is it just extended life-span? This is certainly a question with LSD newborn screening. As experience of screening increases the issues are changing—some have largely been overcome, and some are being newly observed. I have chosen the examples of screening for Krabbe and Pompe disorders to illustrate this clearly. Other disorders now screened for do also illustrate some of these points, but overall the outlook in screening for LSD seems positive.

## 2. Newborn Screening for Pompe Disease (Glycogen Storage Disease Type II)

Pompe disease has a broad clinical spectrum—babies with infantile onset develop hypotonia and hypertrophic cardiomyopathy in the first months of life, and usually die in the first 12 months of cardio-respiratory failure or pneumonia. In later onset forms, which are more common, mainly skeletal muscle is affected, and there are respiratory problems with respiratory failure supervening. Treatment with ERT can ameliorate the clinical features if implemented sufficiently early. This involves frequent (sometimes weekly) infusions, significant immunological problems, and financial costs lifelong. Screening was instituted for Pompe disease in Taiwan in 2005 and has spread, with screening now in more than 11 programmes. It is currently on the recommended list of the American Advisory Committee on Heritable Disorders in Newborns and Children. The Taiwan screening programme reported their experiences of screening 132,500 patients between 2005 and 2007 [11] and more recently have published long-term outcomes in 10 patients with classic infantile-onset disease, all CRIM (cross-reactive immunological material) positive [12]. The 10 patients began ERT treatment at a median of 16 days (6–34 days). All survived, and at a median age of 5 years 3 months (28–90 months) none were ventilated, and cardiac function was generally good. The initial results were impressive, but worrying trends emerged. Motor milestones were initially achieved normally, but 9 of the 10 exhibited weakness later, developing a waddling gait, all had hypernasal speech, and 6 had hearing loss. Seven of nine had white-matter abnormalities on magnetic resonance imaging. It is unclear yet if there will be significant effects on cognition, but it seems likely. Cognitive problems were not apparent before screening since most affected babies died very early. Current management is an increased dosage of ERT, and it is too soon to assess the effects of this. These results are similar to several reports of patients treated early—some clear advantages but evidence of progression of disease (e.g., [13]). Ongoing progressive disease is not the only problem in assessing how much benefit there is in early diagnosis, but it is a major concern. It is possible that new therapies may be developed in the future, but at present this is still a progressive disease. Programme performance can be improved by having some second-tier testing and using multivariant pattern recognition [14], but the issue of ongoing progressive disease, and how much and how early is tolerable, needs to be discussed. However, at present, screening for Pompe disease seems overall to be beneficial for early-onset disease when treatment is started extremely early. Benefits are recorded for the more common late-onset disease also, but so far there is no clear evidence supporting very early diagnosis of late-onset cases [15].

## 3. Newborn Screening for Krabbe Disease (Globoid-Cell Leukodystrophy)

Krabbe disease results in demyelination of both central and peripheral nervous systems. In the classical infantile form, babies have symptoms of irritability and feeding problems starting before six months, progressing to hypertonic episodes, opisthotonus, blindness, seizures, and death usually before two years. Cord-blood stem-cell transplantation (HSCT) before symptoms are apparent was initially thought to be very effective [16]. Certainly, if successful, it appears to prolong life, but does not at present prevent later disease progression. Newborn screening was mandated in New York State in 2006 following pressure from parents. The screening system was carefully set up and has been run extremely well. Recently the results of eight years’ screening have been published by Orsini and by Wasserstein and colleagues [17,18]. They make discouraging reading. Almost 2 million babies were screened; 348 were referred for follow-up (0.017%) and 143 were found to be at some risk (low, medium, high). From 2012, only the high and medium risk category were followed by a modified algorithm [17]. By the end of the eighth year, 5 were diagnosed as early-onset disease and 46 were asymptomatic but classed as moderate to high risk for Krabbe disease [18]. Of the confirmed cases all were offered HSCT. One family declined, and that child died. Two died following complications of transplant. Both the children who were transplanted at one and two months respectively have moderate to severe developmental and motor problems. Even more worrying than this dismal record of affected patients is the issue with the patients “at risk” but currently asymptomatic. Orsini and colleagues have published a wealth of molecular information from the screening programme but they say, “Classification of pathogenic versus non-pathogenic variants will take years of follow-up and cannot be accurately distinguished at this time” [17] (p. 243). However, as mentioned above, second-tier tests integrated with pattern recognition of other markers can greatly improve specificity, while in some instances worsening sensitivity for later-onset cases [14], which could be regarded as a plus by some. Certainly, this is true for Krabbe disease screening. Indeed, using psychosine as a second-tier test is part of the currently recommended screening protocol [19]. The New York State and Kentucky programmes, with help from the Mayo Clinic group [14] have worked hard to ameliorate problems of false positive results in Krabbe disease and have provided much valuable information. Even so, at present, harms (including costs of all kinds) seem likely to outweigh the slender benefits. Other papers attest to this, showing improved survival, but ongoing significant disability in most if not all presymptomatically-treated patients [20]. This is a disorder that possibly should not be screened for at present.

## 4. The Two Important Questions

Each LSD has different problems and different issues, but as we brace for molecular screening as a primary test in some form—panels, whole exome screening, or even more—which will greatly complicate things, there seem to be two important questions. Firstly, are we radically altering our concept about the aims of newborn screening? Are we aiming to test babies only for disorders of infancy and childhood and trying to avoid detecting adult-onset cases, or should we embrace finding adult-onset cases, (but need to discuss how to avoid most harm) or should we even test for disorders that are principally adult-onset ones? It is certainly cheaper and more efficient to perform a screening test on newborns, a captive population, rather than later, but is it desirable? Secondly, can we define what is meant by benefit, how much improvement counts as benefit, and who should benefit?

## 5. Mission Creep

Many jurisdictions have adopted overall statements of the aims and purpose of newborn screening. The proposed policy framework for Australia [21] states that, “The aim of newborn bloodspot screening is to improve the health of babies by identifying those at risk of developing a serious condition early, generally before symptoms present, thereby enabling earlier intervention.” Other documents state similar aims. In general, it has been understood that newborn screening should not be for the purpose of identifying adult-onset conditions, but this is nowhere stated. There is also considerable debate as to whether extra conditions should be added to the newborn screening test for reasons other than a mortality or morbidity benefit to the child screened. Benefit to the family, say, relating to future prenatal diagnosis, is one aspect considered, and this might be relevant to universal screening (as opposed to boys only) in X-linked disease. But screening for disease that mainly manifests in adulthood, or for disorders that are at present untreatable but could inform family planning (such as some LSDs), is a big expansion of what has been envisioned until now. The problems at present, with genotype being found an increasingly unreliable indicator of phenotype, makes the identification in infancy of possible adult-onset disease very worrying.

## 6. The Issue of Benefit

The issue of what is a benefit has been less discussed. In LSDs in general it appears that we now have the ability to identify and treat affected patients early, prolonging life. In some disorders this may convert what was a dreadful disorder resulting in early infant death into a relentlessly progressive disorder, the evolution and extent of which we currently do not understand, and the treatment of which is very burdensome and expensive. Is this always in the child’s best interest? On the other hand, if there is no expansion of newborn screening for fear of difficult results there will probably be little progress on truly tackling disorders for which late treatment is too late. What should be done? Certainly, assessment of the public’s views about LSD newborn screening tend to be positive (e.g., [22]) but it will take a while to be sure that there are sufficient benefits in some cases, for example, in Fabry disease [23].

I believe that several areas of newborn screening need to be strengthened and just now this applies particularly to LSD screening:

(1) *Follow-up: *At present many countries do have well-organised systems for assessing disorders for inclusion in screening [24], but often poor follow-up, so that otherwise well-written reports on outcomes which could illuminate the situation can be so deficient in on-going data, through little or no fault of the authors, that it is hard to draw helpful conclusions. The implementation of screening for newly added disorders must have the costs of adequate follow-up included and the type of follow-up required should be agreed upon, preferably on a national basis. Nearly always with a new screening programme unexpected problems occur and agreed follow-up protocols will need modifications to capture new findings. For newly included disorders, all screening programmes should as far as possible report results after a reasonable interval. This Journal could perhaps be the best conduit for most of such reports which would be very helpful on an international level.
(2) *Screening programmes not recommended nationally: *When screening programmes are undertaken due to local mandate but are not recommended nationally they should be explicitly identified as pilot programmes with planned research, including re-evaluation after an interval.
(3) *Progressive disease despite treatment: *This could be a vanishing issue if new treatments are developed, such as gene-therapy [25], but at present it is a very difficult problem, and we do not yet have enough information about what to expect. Information for parents about the range of outcomes in specific disorders needs updating as new information becomes available. Parents of affected infants must be given accurate, balanced, and unbiased information based on current knowledge, and should be told that one option is not to have treatment for their baby.
(4) *Research is a necessary part of newborn screening as are properly planned pilot programmes:* Pilot programmes are vital and must not become “screening by stealth”, but be organised as research programmes, perhaps only over a defined time. This is a difficult issue as stopping any screening is problematic.
(5) *Costs:* We should not be afraid of robust engagement with pharmaceutical companies on the issue of costs of treatment.

The rapid developments in technology are greatly improving diagnosis and treatment which makes this is a very exciting time to be working in newborn screening. There is, however, an increased need to proceed with caution, so as to ensure that benefits are as great, and harms as few, as possible.
